# Retraction Note: The novel long non-coding RNA LATS2-AS1-001 inhibits gastric cancer progression by regulating the LATS2/YAP1 signaling pathway via binding to EZH2

**DOI:** 10.1186/s12935-022-02744-2

**Published:** 2022-10-14

**Authors:** Dan Sun, Ying Wang, Huan Wang, Yan Xin

**Affiliations:** 1 Laboratory of Gastrointestinal Onco-Pathology, Cancer Institute and General Surgery Institute, The First Affiliated Hospital of China Medical University, No. 155 Nanjing North Street, Heping District, 110001 Shenyang, China; 2 Department of Oncology, Hanzhong Central Hospital, 723000 Hanzhong, China

The Editors in Chief has retracted this article because of irregularities in Fig. 4 A and G. In Fig. 4 A the BGC823 and NC/BGC823 panels for 0 h appear to overlap and the BGC823 and NC/BGC823 panels for 24 h appear to overlap. In Fig. 4G the BGC823 and NC/BGC823 panels appear to overlap and the SGC7901 and NC/SGC7901 panels appear to overlap. The study had also not received sufficient ethics approval. None of the authors agree to this retraction.

